# Diagnostics and Therapy for Malignant Tumors

**DOI:** 10.3390/biomedicines12122659

**Published:** 2024-11-21

**Authors:** Chung-Che Tsai, Chun-Yu Wang, Hsu-Hung Chang, Phebe Ting Syuan Chang, Chuan-Hsin Chang, Tin Yi Chu, Po-Chih Hsu, Chan-Yen Kuo

**Affiliations:** 1 Department of Research, Taipei Tzu Chi Hospital, Buddhist Tzu Chi Medical Foundation, New Taipei City 231, Taiwan; chungche.tsai@gmail.com (C.-C.T.); chuanhsin032484@gmail.com (C.-H.C.); tintin4125@gmail.com (T.Y.C.); 2 Department of Dentistry, Taipei Tzu Chi Hospital, Buddhist Tzu Chi Medical Foundation, New Taipei City 231, Taiwan; wangchunyu0313@gmail.com; 3 Division of Nephrology, Department of Internal Medicine, Sijhih Cathay General Hospital, New Taipei City 221, Taiwan; hhchang@cgh.org.tw; 4 School of Biological Sciences, University of California, San Diego, CA 92093, USA; p6chang@ucsd.edu; 5 Institute of Oral Medicine and Materials, College of Medicine, Tzu Chi University, Hualien 970, Taiwan

**Keywords:** cancer diagnostics, personalized therapy, tumor biomarkers, molecular diagnostics

## Abstract

Malignant tumors remain one of the most significant global health challenges and contribute to high mortality rates across various cancer types. The complex nature of these tumors requires multifaceted diagnostic and therapeutic approaches. This review explores current advancements in diagnostic methods, including molecular imaging, biomarkers, and liquid biopsies. It also delves into the evolution of therapeutic strategies, including surgery, chemotherapy, radiation therapy, and novel targeted therapies such as immunotherapy and gene therapy. Although significant progress has been made in the understanding of cancer biology, the future of oncology lies in the integration of precision medicine, improved diagnostic tools, and personalized therapeutic approaches that address tumor heterogeneity. This review aims to provide a comprehensive overview of the current state of cancer diagnostics and treatments while highlighting emerging trends and challenges that lie ahead.

## 1. Introduction

Malignant tumors, which are characterized by uncontrolled cellular proliferation, invasion, and metastasis, are a major cause of morbidity and mortality worldwide [1]. Despite significant advancements in early detection and treatment of malignant tumors, the global burden of cancer continues to rise [2]. This can be attributed to factors such as the aging population, environmental changes, and lifestyle [3]. Modern oncology is advancing rapidly with the introduction of personalized medicine, immunotherapy, and novel diagnostic methods; however, challenges remain in ensuring equitable access to these technologies and translating scientific breakthroughs into clinical practice [4]. This review provides a detailed analysis of conventional and emerging diagnostic and therapeutic modalities in oncology.

## 2. Pathophysiology of Malignant Tumors

The development of malignant tumors, or carcinogenesis, is a multistep process involving genetic mutations, epigenetic alterations, and interactions between cancer cells and their microenvironment [5]. Key processes include apoptosis evasion, sustained angiogenesis, tissue invasion, and metastasis [6]. Genetic mutations in oncogenes (e.g., *RAS* and *MYC*) and tumor suppressor genes (e.g., *TP53* and *BRCA1*) are often the driving factors [7]. Beyond these characteristics of malignant tumors mentioned earlier, immune evasion and metabolic reprogramming make cancer cells more aggressive within the human body. Cancer cells evade immune system attacks, including those from T cells and natural killer (NK) cells, which support their survival [8]. In colon cancer, a scarcity of killer lymphocytes is linked to poorer prognosis compared to patients with abundant T cells [9]. Additionally, the reprogramming of energy metabolism enables cancer cells to adapt to substantial alterations in the tumor microenvironment, which are driven by growth factors and hypoxia-induced factors [8]. Through the Warburg effect, cancer cells rely on fermentation to generate energy and sustain their high proliferation capacity [10]. Thus, these two characteristics, avoiding immune destruction and deregulating cellular energetics, can further facilitate tumor progression [11].

## 3. Diagnostic Techniques in Malignant Tumors

Accurate and early diagnosis is crucial for improving cancer outcomes [12]. Diagnostic approaches such as imaging techniques and molecular diagnostics have evolved significantly, incorporating both traditional and advanced techniques [13].

### 3.1. Imaging Techniques

Computed tomography (CT) and magnetic resonance imaging (MRI) remain as the cornerstone imaging techniques for tumor detection and staging. They offer high-resolution imaging, enabling detailed tumor characterization [14]. Paudyal et al. highlighted the increasing utility of artificial intelligence (AI) in enhancing tumor detection, segmentation, and monitoring, contributing to more precise and personalized cancer treatment. This review discusses how AI can streamline clinical workflows, improve image quality, and address the need for quantitative metrics beyond traditional lesion size measurements. However, it also identifies significant challenges, including data accuracy, bias, and the integration of AI tools into clinical practice in the near future [15]. Positron emission tomography (PET) scans, often combined with CT, are valuable for detecting metastasis by highlighting areas of high metabolic activity typical of cancer cells [16]. PET is particularly useful for monitoring responses to therapy [17]. Illimoottil and Ginat explored the application of deep learning (DL) to improve the diagnosis, treatment, and prognosis of head and neck cancers using imaging techniques such as MRI, CT, and PET. It highlights how advanced DL models, such as convolutional neural networks, generative adversarial networks, and transformer models, can enhance tumor detection, segmentation, and outcome prediction. These methods address some limitations of traditional imaging, including the subjectivity and variability in human interpretation [18]. Ultrasound and endoscopy have been reported to be useful medical technologies for certain cancers, such as pancreatic and gastrointestinal cancers, and they allow real-time imaging coupled with biopsy capabilities [19,20]. Qi et al. described the development and application of a novel endoscopic imaging technique called surgical polarimetric endoscopy (SPE). SPE uses polarized light to enhance the detection of laryngeal cancer, providing greater contrast between cancerous and healthy tissues than with traditional white light endoscopy. By measuring the differences in light polarization, SPE offers real-time, high-definition images that significantly improve cancer detection accuracy, particularly for early-stage and precancerous lesions that are difficult to distinguish using standard methods [21].

Therefore, CT and MRI remain essential for tumor detection and staging, providing high-resolution imaging that allows for detailed characterization of malignancies. The integration of AI and DL is transforming oncological imaging, enhancing tumor detection, segmentation, and monitoring while also addressing limitations such as variability in human interpretation. PET, particularly in combination with CT, plays a critical role in identifying metastases and monitoring therapeutic responses. Emerging techniques such as SPE offer promising advancements in early cancer detection by significantly improving the contrast between cancerous and healthy tissues. Despite this progress, challenges such as data accuracy, bias, and the integration of AI into clinical practice must be addressed to fully realize the potential of these technologies.

### 3.2. Molecular Diagnostics

Molecular diagnostics have become a cornerstone in the detection, classification, and management of cancer [22]. This approach uses genomic, transcriptomic, proteomic, and epigenetic alterations in cancer cells to provide precise and individualized insights into the biology of the disease [23,24]. Rapid advancements in molecular diagnostics have enabled earlier detection, more accurate prognosis, and the development of personalized treatment strategies [25]. This section covers the key molecular diagnostic tools currently used in oncology, including tumor biomarkers, tissue biopsies, and liquid biopsies.

#### 3.2.1. Tumor Biomarkers for Diagnosis

Tumor biomarkers play a critical role in cancer diagnosis by providing measurable indicators of the presence or progression of malignancies [26]. These biomarkers are typically found in blood, urine, or tissues and are produced either by the tumor itself or by the body in response to cancer [27]. Identification and monitoring of these biomarkers have revolutionized cancer diagnostics by enabling early detection, improving prognosis, and guiding therapeutic decisions [28]. Although blood-detected biomarker levels need to be combined with other specific tests, including magnetic resonance imaging, transvaginal ultrasound, computed tomography, and additional biomarkers to achieve greater accuracy, they still hold credible diagnostic ability for specific cancers. Tumor biomarkers can be classified into several categories based on their molecular nature and diagnostic utility, including proteins, nucleic acids (genetics), lipids, metabolites, long non-coding RNAs (lncRNAs), and microRNAs (miRNAs) [27]. In this section, we summarize the various types of tumor biomarkers (Table 1, Table 2 and Table 3) and their applications in clinical oncology.

**Table 1:** Potential biomarkers for diagnosed cancer type.

Molecule	Biomarker	Diagnostic Value	Diagnosed Cancer Type	Reference
Protein	PSA	2–10 ng/mL	Prostate cancer	[29,30]
	CA-125	>30 U/mL	Ovarian cancer	[31,32]
	CEA	5–10 μg/L	Colorectal cancer	[33,34]
	AFP	>20 ng/mL	Hepatocellular carcinoma and germ cell tumor	[35,36]
Nucleic acid	*BRCA1*, *BRCA2*	18 ng/mL	Breast cancer and Ovarian cancer	[37,38]
	*EGFR*	197 copies/μL	Non-small cell lung cancer	[39,40]
	*KRAS*	>0.8 ng/μL (pancreatic cancer); 5450 alleles/mL (lung cancer)	Colorectal cancer, pancreatic cancer, and lung cancer	[41,42,43]
	Methylated *SEPT9*	16 copies/mL	Colorectal cancer	[44,45]
	Methylated *MGMT*	25.2 ng/mL	Glioblastoma	[46,47]
Lipid	LPA	3.5 μmol/L (ovarian cancer); 0.1 μmol/L (breast cancer); 2.58 nmol/mL (prostate cancer)	Ovarian cancer, breast cancer, and prostate cancer	[47,48,49,50]
	PC	>0.28 μmol/L	Breast cancer, liver cancer, and colorectal cancer	[51,52,53,54]
	S1P	75–1100 nM	Breast cancer, ovarian cancer, and colorectal cancer	[55,56,57]
	Ceramide	0.00744 pmol/mg	Breast cancer	[58,59]
	27-HC	0.31 μM	Breast cancer	[60,61]
	Cholesterol ester	No report	Prostate cancer and glioblastoma	[62,63]
	FFA	>0.4 mmol/L	Breast cancer and prostate cancer	[64,65,66]

Protein biomarkers are some of the most commonly used biomarkers in clinical practice. They are typically found in blood or tissues and can indicate the presence of cancer [67]. Prostate-specific antigen (PSA) is a well-known biomarker for the early detection of prostate cancer. Elevated PSA levels (2–10 ng/mL) in the blood can indicate the presence of prostate cancer, although benign conditions such as prostatitis or benign prostatic hyperplasia can also elevate PSA levels [29]. CA-125 is widely used to detect ovarian cancer. Elevated CA-125 levels (>30 U/mL) can indicate the presence of ovarian cancer; however, they are also elevated in other conditions, such as endometriosis, making it more useful for monitoring treatment response rather than for early detection [31]. Carcinoembryonic antigen (CEA) is primarily used as a biomarker for colorectal cancer, with levels ranging from 5 to 10 μg/L, but it can also be elevated in other cancers, such as breast, lung, and pancreatic cancers. It is typically used to monitor the disease progression and recurrence after treatment [33]. Alpha-fetoprotein (AFP) is a diagnostic marker for liver cancer (hepatocellular carcinoma) and germ cell tumors. AFP levels above 20 ng/mL in the blood can indicate the presence of these cancers, particularly in patients with underlying liver diseases [35].

Genetic tumor biomarkers are characterized by changes in DNA or RNA that indicate the presence of cancer. Genetic biomarkers provide insights into the molecular alterations driving tumor growth and can guide targeted therapies [68]. Mutations in the breast cancer gene 1 (BRCA1) and breast cancer gene 2 (BRCA2) genes significantly increase the risk of breast and ovarian cancers [69]. Testing for these mutations can guide preventive measures and inform treatment decisions, particularly for poly ADP-ribose polymerase inhibitors [37]. Epidermal growth factor receptor (EGFR) mutations are commonly found in non-small cell lung cancer [39]. Detecting these mutations can help select patients for targeted therapy with EGFR inhibitors such as erlotinib and gefitinib [41]. Kirsten rat sarcoma virus mutations are commonly observed in colorectal, pancreatic, and lung cancers [41]. Kirsten rat sarcoma virus mutations are often associated with resistance to certain targeted therapies such as anti-EGFR monoclonal antibodies in colorectal cancer [70]. Moreover, emerging biomarkers are used to assess genetic predispositions (BRCA mutations) and guide treatment options (human EGFR 2 in breast cancer and EGFR in lung cancer) [71]. Moreover, blood-based epigenetic biomarkers are used to reduce sampling invasiveness, simplify sampling procedures, and detect changes in gene expression without altering the DNA sequence itself, often through mechanisms such as DNA methylation or histone modification [72,73]. Methylated septin 9 DNA is a biomarker used in the detection of colorectal cancer through blood tests [44]. It is particularly useful for screening individuals at risk of colorectal cancer and offers a non-invasive alternative to colonoscopy [74]. Methylation of the O6-methylguanine-DNA methyltransferase gene promoter is a predictive biomarker of glioblastoma [46]. Tumors with this epigenetic alteration respond better to alkylating agents, such as temozolomide, because methylation silences the DNA repair gene, making the tumor more susceptible to DNA-damaging therapies [75]. These biomarkers will pave the way for more personalized and effective cancer treatments.

Lipids play critical roles in cancer biology, serving not only as structural components of cell membranes but also as signaling molecules that regulate cellular processes such as proliferation, apoptosis, and metabolism [76]. Aberrant lipid metabolism is a hallmark of cancer, and changes in the lipid composition and profile have been increasingly recognized as potential biomarkers for cancer diagnosis, prognosis, and therapeutic targeting [77]. Lipid-based tumor biomarkers offer promising insights into the metabolic alterations that occur in cancer cells and can serve as non-invasive diagnostic tools through blood or tissue analysis [76]. A discussion of the clinical applications of lipid biomarkers in cancer is presented in this section. Lipids are a diverse group of molecules. Different classes of lipids, including phospholipids, sphingolipids, fatty acids, and cholesterol derivatives, have been identified as potential biomarkers of various cancers [78]. Phospholipids, which constitute the structural backbone of cell membranes, show altered profiles in cancer cells due to changes in membrane synthesis and cellular signaling [79]. Lysophosphatidic acid (LPA) is a bioactive phospholipid that acts as a signaling molecule involved in cell growth, survival, and migration [80]. Increased levels of LPA have been detected in ovarian (3.5 μmol/L), breast (0.1 μmol/L), and prostate cancers (2.58 nmol/mL). It is particularly significant in ovarian cancer, where LPA levels in the plasma and ascitic fluid are often elevated, making it a potential biomarker for early detection [48,49]. Phosphatidylcholines (PCs) are the most abundant phospholipids in the cell membrane [81]. Alterations in PC metabolism, especially in levels of certain PC species, have been linked to breast, liver, and colorectal cancers. These alterations reflect the metabolic reprogramming of cancer cells to support rapid cell growth and membrane biogenesis [51,52,53]. Sphingolipids are involved in regulating cell death, proliferation, and differentiation. The dysregulation of sphingolipid metabolism is associated with cancer progression [82,83]. Sphingosine-1-phosphate (S1P) is a signaling sphingolipid that promotes tumor growth, angiogenesis, and metastasis by interacting with the S1P receptors on cancer cells. Elevated levels of S1P, ranging from 75 to 1100 nM, have been detected in breast, ovarian, and colorectal cancers, and are considered potential biomarkers of cancer progression and metastasis [55,56]. Ceramides, a subclass of sphingolipids, are involved in apoptotic signaling pathways [84]. During carcinogenesis, the balance between ceramides and S1P is often disrupted, which leads to increased cell survival [85]. Reduced ceramide levels are linked to chemotherapy resistance in certain cancers, such as breast cancer, making ceramide a biomarker of therapeutic response [58].

Cholesterol and its metabolites play essential roles in cancer cell membrane structure and signaling pathways [86]. Oxysterols are oxidized derivatives of cholesterol involved in the regulation of immune responses and cell proliferation [87]. Elevated levels of specific oxysterols, such as 27-hydroxycholesterol (27-HC), are associated with breast cancer progression, particularly in estrogen receptor-positive breast cancer, where they can mimic estrogen and promote tumor growth [60]. Increased levels of cholesterol esters have been found in aggressive forms of cancer, such as prostate cancer and glioblastoma [62,63]. Cholesterol ester accumulation reflects altered lipid metabolism in cancer cells driven by increased uptake and synthesis of cholesterol to support membrane production and oncogenic signaling [88]. Furthermore, fatty acids, both free and as components of complex lipids, are essential for cancer cell proliferation and survival [89]. Cancer cells often exhibit elevated levels of free fatty acids (FFAs) due to enhanced lipolysis and de novo fatty acid synthesis [90]. Elevated levels of certain FFAs above 0.4 mmol/L, such as palmitic and oleic acids, have been linked to breast and prostate cancers [64,65]. FFAs can be detected in blood samples and may serve as non-invasive biomarkers for cancer diagnosis and monitoring [91,92]. Alterations in the ratio of omega-6 to omega-3 polyunsaturated fatty acids (PUFAs) have been implicated in cancer progression [93]. Increased levels of omega-6 PUFAs, such as arachidonic acid, have been associated with inflammation and cancer growth, whereas omega-3 PUFAs, such as eicosapentaenoic acid, have been shown to have anti-inflammatory and anti-tumorigenic effects [94,95,96]. Taken together, the ratio of these fatty acids could serve as a potential biomarker of cancer risk and progression.

Metabolites are small molecules generated or consumed during metabolic processes. Metabolism encompasses both the breakdown of substances to produce energy (catabolism) and the synthesis of complex molecules (anabolism). Dysregulated cellular metabolism is a hallmark of cancer, leading to elevated levels of normal metabolites or the production of abnormal ones. These altered metabolite levels can serve as biomarkers to help predict cancer. Tumor cells can modify their metabolic pathways to support biosynthesis and meet energy demands, adapting to the influence of oncogenic mutations and changes in tumor suppressor functions [97,98]. To date, a significant body of evidence has shown that different types of metabolites exhibit abnormal levels in the blood (Table 2). However, identifying specific cancers based on circulating metabolite levels remains a major challenge in developing effective non-invasive diagnostic biomarkers.

**Table 2:** Metabolites as biomarkers for various cancer types.

Cancer Type	Metabolite	Reference
Breast cancer	Creatinine (↑), sarcosine (↑), 5-oxoproline (↑), L-phenylalanine (↑), glycoursodeoxycholic acid (↑), glycochenodeoxycholic acid, (↑) tauroursodeoxycholic acid (↑), 1-methylnicotimanide (↑), octanoic acid (↑), dodecanoylcarnitine (↑), L-acetylcarnitine (↑), docosahexaenoic acid (↑)	[99]
Ovarian cancer	arabitol (↑), maltose (↑), maltotriose (↑), raffinose (↑), mannitol (↑), demethylphylloquinone (↓), ganglioside (↑), N-formylkynurenine (↑), histidine (↓), citrulline (↓), citrate (↓), lysine (↑)	[100]
Prostate cancer	Sarcosine (↑), kynurenine (↑), choline (↑), spermine (↑), citrate (↑), myo-inositol (↑), fructose (↑)	[101,102]
Pancreatic cancer	creatine (↑), inosine (↑), beta-sitosterol (↑), sphinganine (↑), glycocholic acid (↑), acetylcarnitine (↑), glutamine (↑), glutamic Acid (↓), symmetric dimethylarginine (↑), hexoses (↓)	[103,104]
Gastric cancer	Glucose (↓), lactate (↓), fumaric acid (↓), citrate (↑), α-ketoglutarate (↑), succinate (↓), pyruvic acid (↓), valine (↑), tryptophan (↓), leucine (↓), histidine (↓), glutamine (↑), gondoic acid (↑), palmitoleic acid (↑), cervonic acid (↑)	[105]
Colon cancer	5-hydroxytryptamine (↓), fumarate (↓), 4-hydroxystyrene (↓), hydroquinone (↓), cholic acid (↓), 2-hydroxy-3-methylpentanoic acid (↓), xanthosine (↑), sphinganine (↑), octenedioate (↑), β-hydroxybutyrate (↑), 2-oxobutanoic acid (↑)	[106]
Bladder cancer	Isobutyrate (↑), pyroglutamate (↑), propionate (↑), choline (↑), acetate (↑)	[107]
Acute myeloid leukemia	2-hydroxyglutarate (↑)	[108]
Thyroid cancer	Choline (↑), glucose (↑), mannose (↑), pyruvate (↑), 3-hydroxybutyric acid (↑), valine (↓), tyrosine (↓), proline (↓), lysine (↓), leucine (↓), gamma-aminobutyric acid (↑), aminooxyacetic acid (↑), 4-deoxypyridoxine (↑); pyroglutamic acid (↓)	[109]
Liver cancer	Putrescine (↑), cadaverine (↑), spermidine (↑), agmatine (↑), lysine (↑), arginine (↑), *S*-adenosyl-l-methionine (↑), *N*-acetylspermine (↑), *N*-acetylspermidine (↑), *γ*-aminobutyric acid (↑)	[110]
Lung cancer	Putrescine (↑), cadaverine (↑), spermidine (↑), agmatine (↑), ornithine (↑), lysine (↑), arginine (↑), *S*-adenosyl-l-methionine (↑), *γ*-aminobutyric acid (↑)	[110]

↑: upregulation; ↓: downregulation.

In addition to these regular molecules as potential biomarkers for cancer diagnosis, lncRNAs and miRNAs have been found to exhibit differential expression between healthy individuals and cancer patients. lncRNAs are a type of RNA molecule defined as transcripts longer than 200 nucleotides that do not code for proteins but play crucial roles in regulating gene expression and cellular processes, including growth, differentiation, migration, and apoptosis. lncRNAs regulate the expression of oncogenes and tumor-suppressor genes in normal cells, and their dysregulation can promote tumorigenesis [111]. The levels of lncRNAs are associated with different types of cancer compared to normal tissue (Table 3). For instance, HOTAIR promotes metastasis in breast cancer by modifying chromatin states [112]. Conversely, the loss or downregulation of certain lncRNAs, such as MEG3, can disrupt critical cell growth regulation, contributing to cancer development [113]. Similar to lncRNAs, miRNAs, non-coding RNA molecules containing 21–23 nucleotides, have a role in cancer-associated biological processes [114]. As shown in Table 3, different miRNAs are associated with various types of cancer progression. Although the levels of both lncRNAs and miRNAs can change in the blood of cancer patients, a challenge remains in determining the specificity of these RNAs for diagnosing specific cancer types.

**Table 3:** lncRNA and miRNA as biomarkers for various cancer types.

Cancer Type	lncRNA	miRNA	Reference
Breast cancer	ZFAS1 (↓), LSINCT5 (↑), LINC00617 (↑), RP11-445H22.4 (↑), BC200 (↑), UCA1 (↑), SRA (↑), HOTAIR (↑)	miR-126 (↓), miR-335 (↓), miR-199a (↓), Jet-7c (↓), Jet-7d (↓), miR-589 (↑), miR-425 (↑), miR-21 (↑), miR-34a (↑), miR-106a (↑), miR-195 (↑), Jet-7a (↑)	[115,116,117,118,119,120,121,122,123,124,125]
Glioma	TSLC1-AS1 (↓), ADAMTS9-AS2 (↓), MDC1-AS (↓), TUG1 (↓), ROR (↓), CACS2 (↓), GAS5 (↓), MEG3 (↓), XIST (↑), CRNDE (↑), MALAT1 (↑), HOTAIR (↑), HOXA11-AS (↑), Linc-POU3F3 (↑), ATB (↑), AB073614 (↑), H19 (↑), SPRY4-IT1 (↑)	miR-29 (↓), miR-128 (↓), miR-205 (↓), miR-125b (↓), miR-122 (↓), miR-451a (↓), miR-203 (↓), miR-219-5p (↑), miR-21 (↑), miR-376c (↑), miR-210 (↑), miR-301a (↑), miR-454-3p (↑)	[126,127,128,129,130,131,132,133,134,135,136,137,138,139,140,141,142,143,144,145]
Colorectal cancer	LOC554202 (↓), PVT1 (↑), H19 (↑), AFAP1-AS1 (↑), MALAT1 (↑), CCAT1-L (↑), PCAT-1 (↑)	miR-10b (↓), miR-155 (↓), miR-29a (↑), miR-92a (↑), miR-141 (↑), miR-221 (↑), Jet-7a (↑)	[124,146,147,148,149,150,151,152,153,154,155]
Prostate cancer	PTENP1 (↓), PCA3 (↑), PCAT5 (↑), PCAT18 (↑), PRNCR1 (↑), MALAT1 (↑), PCAT-1 (↑),	miR-155 (↓), miR-21 (↑), miR-141 (↑), miR-221 (↑), miR-375 (↑), Jet-7a (↑)	[124,156,157,158,159,160,161,162,163,164]
Gastric cancer	LINC00152 (↑), LSINCT-5 (↑), H19 (↑), PVT1 (↑),	Jet-7a (↓), miR-21 (↑), miR-106a (↑), miR-106b (↑), miR-17-5p (↑), miR-1 (↑), miR-34a (↑), miR-20 (↑), miR-27a (↑),	[165,166,167,168,169,170]
Pancreatic cancer	HULC (↑), HOTAIR (↑)	miR-155 (↑), miR-21 (↑), miR-196a (↑), miR-210 (↑), miR-155 (↑), miR-200a (↑), miR-200b (↑),	[171,172,173,174,175]
Lung cancer	MALAT1 (↓), MEG3 (↓), UCA1 (↑), AFAP1-AS1 (↑), HOTAIR (↑), CCAT2 (↑), MVIH (↑), LCAL1 (↑), LUADT1 (↑)	miR-30e-3p (↓), Jet-7f (↓), miR-1 (↓), miR-17-5p (↓), miR-27a (↓), miR-106a (↓), miR-146 (↓), miR-155 (↓), miR-221 (↓), miR-499 (↓), Jet-7a (↓), miR-21 (↑), miR-25 (↑), miR-29c (↑), miR-30d (↑), miR-223 (↑), miR-486 (↑)	[113,119,176,177,178,179,180,181,182,183,184,185,186,187]
Hepatocellular cancer	PRAL(↓), MALAT1 (↑), HOTAIR (↑), RP11-160H22.5 (↑), XLOC_014172 (↑), LOC149086 (↑), BANCR (↑), SNHG3 (↑), MVIH (↑), ANRIL (↑), HULC (↑)	miR-92a (↓), miR-21 (↑), miR-122 (↑), miR-223 (↑), miR-500 (↑), miR-885-5p (↑)	[188,189,190,191,192,193,194,195,196,197,198,199,200]
Oral cancer	NEAT1 (↑), UCA1 (↑), HOTAIR (↑)	miR-24 (↑), miR-31 (↑)	[201,202,203,204,205]
Acute myeloid leukemia	Wt1-as (↑)	miR-92a (↓)	[206,207]
Cervical cancer	CCHE1 (↑), HOTAIR (↑), CCAT2 (↑)	miR-1284 (↓), miR-573 (↓), miR-433 (↓), miR-424-5p (↓), miR-361-5p (↓), miR-383-5p (↓), miR-335-5p (↓), miR-874 (↓), miR-132 (↓), miR-411 (↓), miR-545 (↓), miR-143 (↓), miR-107 (↓), miR-1 (↓), miR-195 (↓), miR-31 (↑), miR-224 (↑), miR-92a (↑), miR-200a (↑), miR-96-5p (↑), miR-199b-5p (↑),	[208,209,210,211]
Melanoma	CASC15 (↑), SPRY4-IT1 (↑)	miR-10b (↓), miR-155 (↓)	[124,212,213,214]
Bladder cancer	H19 (↑), UCA1 (↑)	miR-21 (↑), miR-210 (↑), miR-29c (↓), miR-124 (↓), miR-29c (↓), miR-214 (↓), miR-29c (↓)	[215,216,217]

↑: upregulation; ↓: downregulation.

#### 3.2.2. Tissue and Liquid Biopsies for Tumor Diagnosis

Tissue biopsy is a critical component in the diagnosis and management of tumors. It involves the extraction of a tissue sample from a suspicious lesion or mass for histological examination. This process is essential to confirm the presence of cancer, determine its type, and guide treatment decisions. Biopsies provide critical information about a tumor’s histological and molecular characteristics, enabling oncologists to make informed decisions regarding treatment and management [218].

Liquid biopsies that analyze biomarkers from blood and other body fluids to detect cancer non-invasively are growing techniques for transforming cancer diagnosis and management [219]. Although traditional methods, such as tissue biopsies, remain the gold standard, liquid biopsies offer significant advantages, including being less invasive, more easily repeatable, and potentially more cost-effective. However, the current evidence on liquid biopsy technologies often lacks the sensitivity required to detect early-stage cancers [220]. Additionally, liquid biopsy analysis involves the identification of biomarkers for early cancer diagnosis, prognosis, therapeutic prediction, and follow-up by isolating circulating tumor cells, circulating tumor DNA, extracellular vesicles, and tumor-educated platelets from body fluid samples and analyzing their molecular characteristics [221,222].

#### 3.2.3. Integration of Histopathology, Genomics, and Big Data for Personalized Cancer Diagnosis

Histopathology has been a crucial tool for cancer diagnosis and prognosis for over a century. It reveals key features for assessing cancer progression, including molecular insights and characteristics such as nuclear atypia, cell type, disease development, mitotic activity, genetic alterations, cell viability, and tissue architecture [223,224]. Integrating cytological details and complex tissue patterns helps to classify and grade lesions. Histopathology is essential for a precise diagnosis by integrating genomic information [225]. The identification of specific genetic events in tumors has been driven by the rapid growth of molecular diagnostics such as polymerase chain reactions and immunohistochemistry [225].

Molecular diagnostics are increasingly required to assist in diagnosing various tumors, transitioning from the study of tumor pathogenesis to applications in clinical laboratories [225]. To date, several tumor-related genes have been shown to be involved in cancer development (Table 4). For example, the identification of BRCA1 and BRCA2 mutations in breast cancer, mutL homolog 1, mutS homolog 6, and mutS homolog 6 mutations in colon cancer, and the RB1 mutation in retinoblastoma is useful for understanding cancer progression in relation to different prognoses and treatments [226,227,228]. However, point-by-point identification of tumor-related genes is not applicable to all patients, and the cost of personalized molecular identification is high. Thus, the integration of molecular profiling information with big data for personalized diagnosis is currently ongoing. To achieve this objective, the following tasks must be completed.

**Table 4:** Cancer types are caused by inherited mutant genes.

Cancer Type	Inherited Genes	Reference
Breast cancer	*BRCA1*, *BRCA2*, *tumor protein P53* (*TP53*), *phosphatase and tensin homolog* (*PTEN*), *mutY DNA glycosylase* (*MUTYH*), *serine/threonine kinase 11* (*STK11*)	[229,230,231,232,233]
Ovarian cancer	*BRCA1*, *BRCA2*, *human mutL homolog 1* (*MLH1*), *mutS homolog 2* (*MSH2*), *mutS homolog 6* (*MSH6*), *postmeiotic segregation increased 2* (*PMS2*), *MUTYH*, *STK11*, *PTEN*	[229,231,232,233,234]
Prostate cancer	*BRCA2*, *MLH1*, *MSH2*, *MSH6*, *PMS2*	[235,236]
Pancreatic cancer	*BRCA1*, *BRCA2*, *adenomatous polyposis coli* (*APC*), *STK11*, *multiple endocrine neoplasia type 1* (*MEN1*), *cyclin-dependent kinase inhibitor 2A* (*CDKN2A*)	[237,238,239]
Gastric cancer	*BRCA1*, *BRCA2*, *MLH1*, *MSH2*, *MSH6*, *PMS2*, *APC*, *STK11*, *MEN1*	[240,241,242,243,244]
Colon cancer	*MLH1*, *MSH2*, *MSH6*, *PMS2*, *PTEN*, *TP53*, *STK11*	[230,231,233,245]
Bladder cancer	*MLH1*, *MSH2*, *MSH6*, *PMS2*, *MUTYH*	[232,246]
Gallbladder cancer	*MLH1*, *MSH2*, *MSH6*, *PMS2*, *TP53*	[247]
Womb cancer	*MLH1*, *MSH2*, *MSH6*, *PMS2*, *PTEN*, *MUTYH*, *TP53*	[248,249,250]
Glioblastoma	*TP53*, *STK11*	[230,233]
Bone cancer	*TP53*	[251]
Acute myeloid leukemia	*TP53*	[252]
Soft tissue sarcoma	*TP53*, *STK11*	[251]
Melanoma	*PTEN*, *STK11*	[233,253]
Thyroid cancer	*PTEN*	[231]
Kidney cancer	*PTEN*	[231]
Liver cancer	*APC*	[254]
Retinoblastoma	*retinoblastoma 1* (*RB1*)	[255]
Lung cancer	*TP53*, *STK11*	[230,233]
Esophageal cancer	*TP53*	[230]

Data Collection and Standardization: Histopathological and genomic data must be collected with high-resolution images, digitized, and then stored alongside DNA sequencing data, including mutations and gene expression profiles [223]. Histopathological images are analyzed using image recognition software, and machine learning to identify patterns and disease markers [223]. Finally, both data types must be standardized for integration.

Data Integration: Patient-specific histopathology images and genomic profiles are integrated into a unified framework for comprehensive analysis. This allows us to examine how genetic alterations manifest as tissue-level abnormalities. Machine learning models, including DL networks, can analyze image and sequence data to identify patterns and predict outcomes [256].

Machine Learning and AI Analysis: AI algorithms can detect cancer types, stages, and genetic mutations from histopathological images, while genomic data can predict outcomes based on mutations and gene expression [257]. Big data improve the accuracy of predictive models, enabling AI to correlate histopathological features with genetic mutations to forecast patient prognosis and treatment response.

Personalized Diagnosis and Treatment: Integration of genomic and histopathological data enables clinicians to identify cancer patterns and predict effective treatments for individual patients. Big-data-powered clinical decision support systems provide real-time diagnosis and treatment recommendations based on similar historical cases [258].

Research and Drug Development: Integrating big data from histopathology and genomics identifies novel biomarkers for early detection and targeted therapy while improving patient selection for clinical trials based on specific genetic profiles or histopathological features [259].

Data Sharing and Collaboration: Uploading anonymized data to global databases accelerates disease discovery and treatment by allowing researchers and healthcare providers to share resources. This collaboration pools data across regions, creating larger datasets that improve the model’s accuracy and robustness [260].

Real-Time Analytics: Real-time analytic tools enable clinicians to process big data quickly, allowing for faster and more accurate assessments of histopathological and genomic data [261]. Applying big data techniques allows the integration of histopathology and genomics into personalized healthcare systems, resulting in more accurate and data-driven diagnoses and treatments [262].

The integration of AI and big data in clinical oncology has transformative potential but is also accompanied by challenges and considerations specific to healthcare environments. AI-driven technologies, particularly machine learning (ML) and deep learning (DL), are increasingly applied in diagnostics and treatment planning [263]. These applications range from enhancing imaging interpretation, such as automated tumor detection and segmentation in MRI or CT scans, to real-time analysis of large-scale genomic data that aids in identifying molecular targets for personalized therapies [15]. Big data can combine patient demographics, imaging, molecular profiles, and outcomes, facilitating advanced predictive analytics that enhance treatment personalization and potentially improve prognostic accuracy [264]. Importantly, the challenges in clinical integration have been reported as associated with data accuracy/reliability, bias/generalizability, infrastructure requirements, and regulatory/ethical considerations. In data accuracy and reliability, clinical settings require high standards of accuracy, as errors in AI-driven diagnostics or treatment recommendations could lead to adverse patient outcomes [265]. AI models depend on high-quality, labeled datasets to achieve these standards, yet variability in data sources, imaging quality, and incomplete patient records can undermine model reliability [266]. In bias and generalizability, AI models trained on biased datasets (e.g., from specific populations) may not perform as well across diverse demographic groups, which could lead to disparities in care quality [267]. Ensuring AI systems are validated on diverse datasets is essential for equitable clinical application [268]. In infrastructure requirements, implementing AI in routine practice necessitates significant infrastructural investment, including data storage solutions for high volumes of imaging and genomic data, computational resources to handle complex analyses, and secure systems to protect patient privacy [269]. Additionally, healthcare providers need training to interpret AI outputs and integrate them into decision making [270]. In regulatory and ethical considerations, regulatory approval for AI models in healthcare remains a lengthy process, often requiring extensive validation studies [271]. Moreover, ethical guidelines for AI usage are still evolving, particularly regarding patient data usage and transparency in AI decision making [272]. Taken together, while AI and big data hold promise for enhancing oncology care, addressing these specific challenges is crucial to their safe, effective, and equitable integration into clinical practice.

## 4. Therapeutic Strategies for Malignant Tumors in Personalized and Targeted Therapies

In recent years, the treatment of malignant tumors has evolved drastically owing to advancements in personalized and targeted therapies [273]. These therapeutic strategies focus on tailoring treatments to the specific genetic, molecular, and environmental factors of each patient’s tumor, thereby increasing treatment efficacy and minimizing side effects. Personalized and targeted therapies have revolutionized cancer care, particularly for cancers that are resistant to traditional treatments such as chemotherapy and radiation [274,275]. This section outlines key approaches and advancements in personalized and targeted therapies for malignant tumors. The following sections explore the current and emerging treatment options.

### 4.1. Precision Molecular Oncology in Personalized Medicine

Precision molecular oncology is at the forefront of personalized medicine and is transforming approaches to cancer diagnosis, treatment, and management [276]. By analyzing the unique molecular characteristics of each patient’s tumor, including genetic, epigenetic, and proteomic alterations, precision oncology enables the development of highly individualized therapeutic strategies tailored to target specific drivers of cancer in each patient [277]. This approach contrasts with traditional “one-size-fits-all” treatments, focusing instead on the underlying biology of each tumor [278]. Next-generation sequencing (NGS) has revolutionized genomics, significantly advancing clinical diagnosis and precision medicine by allowing the rapid, cost-effective analysis of large-scale genetic data. NGS is a powerful tool for identifying disease-causing variants, enabling early and accurate diagnosis of genetic disorders, and facilitating the discovery of novel genes for targeted therapies. The combination of NGS and other omics, AI-driven analysis, would improve genome exploration and analysis for non-invasive monitoring in precision medicine [279,280,281]. Molecular imaging is a non-invasive medical technique that allows for the visualization, characterization, and quantification of biological processes at the molecular and cellular levels within tumors. In the field of theragnostic, molecular imaging offers a vision of how it may be combined with other diagnostic techniques to make cancer treatment more efficient and effective one day [282,283].

### 4.2. Surgery

Surgical resection is often the first-line treatment for localized solid tumors [284]. Advances in minimally invasive surgeries, such as robot-assisted procedures, have improved precision, reduced recovery times, and minimized complications [285]. For some cancers, such as breast cancer, sentinel lymph node biopsies have replaced full lymph node dissection, reducing morbidity [286].

### 4.3. Chemotherapy

Chemotherapy is a cancer treatment that uses cytotoxic drugs to target and kill rapidly dividing cancer cells by interfering with the synthesis of DNA, RNA, or proteins [287]. However, because chemotherapy lacks specificity, it also affects healthy cell growth, leading to side effects [288]. As a result, patients undergoing chemotherapy often experience complications such as anemia, caused by bone marrow suppression, and non-specific toxicities, including fatigue, hair loss, and mouth sores, due to damage to normal rapidly dividing tissues [287].

Several strategies can help minimize these complications. To address anemia, patients may use erythropoiesis-stimulating agents, such as epoetin, darbepoetin, and methoxy polyethylene glycol-epoetin β, which stimulate the bone marrow to produce more red blood cells [289]. For severe anemia, blood transfusions offer an effective way to increase red blood cell counts. If anemia is related to iron deficiency, iron supplements can also be beneficial [290].

For non-specific toxicity, drugs like amifostine can protect certain tissues from the toxic effects of chemotherapy, or symptom-relief medications may be used [291]. Patients can discuss with their doctor the option of optimizing dosage and frequency to balance efficacy and minimize toxicity. If alternative treatments are available, targeted therapy or immunotherapy can specifically target cancer cells, sparing healthy cells and reducing toxicity [292].

Although chemotherapy displays many side effects, combination chemotherapy remains a cornerstone treatment for many cancers, including hematological malignancies (e.g., acute myeloid leukemia) and solid tumors (e.g., breast, lung, and colorectal cancers) [293].

### 4.4. Radiation Therapy

Radiation therapy uses ionizing radiation to kill cancer cells by damaging DNA. Techniques such as intensity-modulated radiation therapy and stereotactic body radiotherapy have increased the precision of radiation delivery, minimizing damage to the surrounding healthy tissues. Proton therapy is an advanced form of radiation that offers even greater precision, particularly for tumors near critical structures (e.g., brain tumors) [294].

### 4.5. Targeted Therapy

Targeted therapies focus on the specific molecular targets that drive cancer growth. These treatments have shown significant success in cancers with known genetic mutations, including tyrosine kinase inhibitors and monoclonal antibodies [295]. For example, drugs such as imatinib for chronic myeloid leukemia and erlotinib for EGFR-mutated lung cancer have transformed treatment paradigms by specifically targeting cancer-driving mutations. Furthermore, antibodies such as trastuzumab (Herceptin) target HER2 in breast cancer, blocking the signaling pathways that promote cell proliferation [296].

### 4.6. Immunotherapy

**Figure 1:**
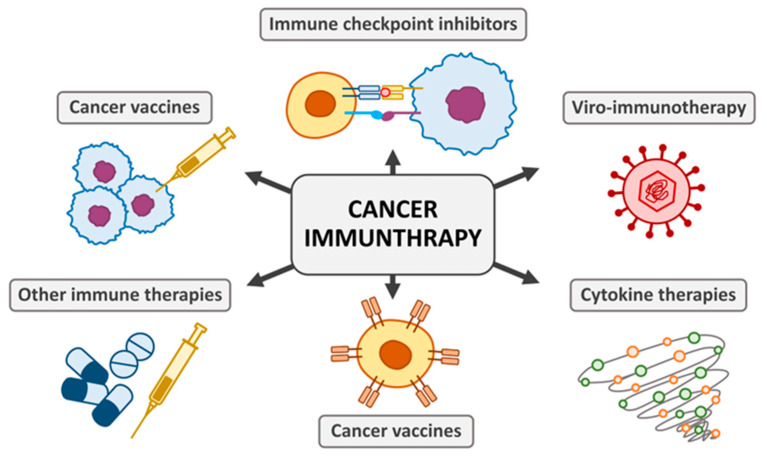
A variety of immunotherapies are employed to treat cancer, including immune-checkpoint inhibitors, cancer vaccines, cytokines, viruses, and adoptive cell transfer.

Cancer therapy has undergone a paradigm shift with the advent of immunotherapy, a treatment modality that leverages the immune system to combat cancer [297] and is summarized in Figure 1. Unlike traditional therapies such as chemotherapy and radiation, which target tumor cells directly, immunotherapy enhances the body’s natural defense mechanisms [292]. The key players in cancer immunology include immune checkpoints, cytokines, and immune cells, such as T cells and natural killer (NK) cells [298]. Immune checkpoints, such as programmed cell death protein 1 (PD-1) and cytotoxic T-lymphocyte-associated antigen 4 (CTLA-4), play critical roles in maintaining immune homeostasis but are exploited by tumors to evade immune responses. Immune checkpoint inhibitors (ICIs), such as pembrolizumab (anti-PD-1) and ipilimumab (anti-CTLA-4), block these pathways, restoring T cell activity against tumor cells [299,300]. In clinical settings, ICIs have transformed the treatment landscape for cancers like melanoma, non-small cell lung cancer, and renal cell carcinoma. However, their efficacy is limited to patients with a pre-existing anti-tumor immune response [301]. The challenge with ICIs involves resistance mechanisms, such as mutations in antigen presentation machinery, and remain as significant barriers. Ongoing research focuses on combining ICIs with other therapies to overcome resistance [302].

There is potential for novel treatments for cancer, autoimmune diseases, and infectious diseases based on cytokine-based therapies [303]. Cytokines are crucial modulators of immune responses. Interleukin-2 (IL-2) and interferon-alpha (IFN-α) are among the first cytokines approved for cancer treatment [304]. It has been reported that high-dose IL-2 therapy promotes the expansion of cytotoxic T cells, leading to positive responses in metastatic melanoma and renal cell carcinoma [305]. On one hand, the advancements of cytokine-based therapy are to develop novel engineered cytokines aimed to enhance specificity and reduce systemic toxicity [306]. For example, IL-15 superagonists are being explored for their ability to activate NK and memory T cells [307]. On the other hand, adoptive cell therapies (ACTs) involve the genetic modification or expansion of immune cells to enhance their anti-tumor activity. Chimeric antigen receptor (CAR) T-cell therapy is the most notable example [308]. CAR T cells are engineered to recognize specific tumor antigens, such as CD19 in B-cell malignancies. Once infused, they proliferate and kill cancer cells [309]. However, challenges include cytokine release syndrome (CRS), neurotoxicity, and limited efficacy in solid tumors. Strategies to improve CAR T-cell trafficking and persistence are under investigation [310].

Cancer vaccines aim to elicit an immune response against tumor-specific or tumor-associated antigens. Examples include dendritic cell-based vaccines, such as sipuleucel-T (Provenge^®^) for prostate cancer [311]. The advantage of cancer vaccine is well known, neoantigen vaccines, personalized based on tumor mutational profiles, have shown promise in early-phase clinical trials [312]. In the near future, combining vaccines with checkpoint inhibitors may synergize immune activation [313]. Interestingly, oncolytic viruses selectively infect and kill tumor cells while stimulating anti-tumor immunity [314]. For example, Talimogene laherparepvec (T-VEC), a herpes simplex virus, has been approved for melanoma [315]. These viruses lyse tumor cells and release tumor antigens, enhancing immune system recognition [316]. Patients with cancer will be able to fully benefit from these emerging agents, which was made possible with T-VEC and other promising oncolytic viruses [317]. Taken together, immunology has revolutionized cancer therapy, providing durable responses in cases where traditional therapies fall short. Thus, continued research is needed to address challenges such as resistance, toxicity, and limited efficacy in certain cancers. The integration of immunotherapies with emerging technologies, such as artificial intelligence and genomics, promises to further enhance their potential.

### 4.7. Hormonal Therapy

Hormonal therapy is primarily used for cancers driven by hormones such as breast and prostate cancers. Drugs, such as tamoxifen (used for breast cancer) and androgen deprivation therapy (used for prostate cancer), block the effects of estrogen and testosterone, respectively, to slow tumor growth [318].

### 4.8. Gene Therapy

Gene therapy is an innovative approach to cancer treatment that involves modifying genetic material to treat or prevent the disease [319]. This strategy targets the underlying genetic and molecular abnormalities that drive cancer progression [320]. Unlike traditional therapies, gene therapy offers the potential for highly specific and long-lasting effects by directly altering the genetic makeup of cancer cells or enhancing the body’s natural defenses against tumors [321]. Many cancers arise from the loss or mutation of tumor suppressor genes, such as TP53 or RB1 [322]. Gene replacement therapy involves delivering functional copies of these genes to cancer cells using vectors, such as adenoviruses or lentiviruses, to restore normal cellular function [323]. Moreover, cancer cells are genetically modified to express a “suicide gene” such as the herpes simplex virus thymidine kinase (HSV-tk) [324]. When exposed to a prodrug like ganciclovir, these cells convert it into a toxic compound, selectively killing the tumor while sparing normal cells [325]. CRISPR-Cas9 technology enables precise editing of cancer-associated genes. It can be used to knockout oncogenes, repair tumor suppressor genes, or introduce genes that enhance immune cell recognition of tumors [326]. Therefore, gene therapy represents a transformative avenue in cancer treatment, offering precision and durability unmatched by conventional therapies. As delivery technologies improve and safety concerns are addressed, gene therapy holds immense potential to redefine the landscape of cancer treatment [327].

Despite significant advancements, cancer treatment still faces critical challenges, including tumor heterogeneity, drug resistance, and adverse effects [328]. Addressing these issues requires innovative strategies that account for tumor complexity, cancer cell adaptability, and patient well-being. Here are some targeted solutions:

1. Tumor Heterogeneity: cancers often consist of multiple subclones with different genetic profiles, leading to resistance to therapy and disease relapse;
  (1) Single-Cell Sequencing and Multi-Omics: applying single-cell sequencing, multi-omics profiling, and spatial transcriptomics enables the identification of diverse cancer cell populations within a tumor, guiding more precise treatment strategies [329];
  (2) Combination Therapy: simultaneously targeting multiple pathways through combination therapies can inhibit the survival of different subclones, reducing the likelihood of resistance [330];
  (3) Adaptive Treatment Approaches: adjusting treatment protocols in response to tumor evolution, known as adaptive therapy, helps control dominant subclones and delays resistance [331].
2. Drug Resistance: resistance to targeted therapies remains a major hurdle, as tumors can adapt by acquiring new mutations, activating alternative pathways, or altering drug targets [332];
  (1) Targeted Therapy Rotation: rotating between targeted therapies before resistance arises may lower the chances of cancer cells adapting to any single treatment [333];
  (2) Next-Generation Inhibitors: developing inhibitors targeting mutations linked to resistance mechanisms can prolong therapy effectiveness [334];
  (3) Targeting Alternative Pathways: drugs designed to block compensatory pathways that tumors activate can provide additional treatment options and prevent adaptation [335].
3. Adverse Effects: Many cancer treatments, including chemotherapy, radiation, and immunotherapy, carry significant side effects, impacting the quality life of patients. Balancing treatment efficacy with safety is a core concern [336].
  (1) Precision Medicine and Biomarkers: tailoring treatments based on biomarkers and genetic profiles allows for more targeted therapy, reducing side effects by matching treatments to individual tumor characteristics [275];
  (2) Prophylactic and Symptom-Management Strategies: integrating supportive care measures to manage side effects, such as antiemetics for chemotherapy-related nausea, improving the quality of life of patients [337];
  (3) Innovative Delivery Systems: utilizing advanced drug delivery systems, like nanoparticle carriers, that selectively target cancer cells can limit harm to healthy cells, reducing adverse effects [338].

These approaches offer a path forward in overcoming the persistent challenges in cancer treatment, aiming to improve both patient outcomes and quality of life.

## 5. Future Directions

The future of oncology lies in the integration of precision medicine, which tailors treatment based on the unique genetic makeup of both the patient and the tumor. Advances in bioinformatics, AI, and machine learning are likely to enhance the ability to analyze complex data, leading to more accurate predictions of treatment responses. Liquid biopsies and real-time monitoring of circulating tumor DNAs are likely to play increasingly important roles in tracking disease progression and treatment efficacy.

## 6. Conclusions

**Figure 2:**
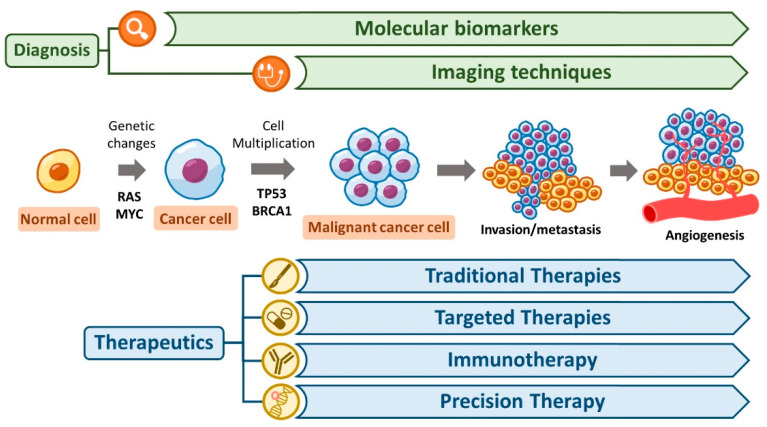
Schematic depiction of diagnostics and therapy for malignant tumors. The cancer development pathway includes three stages. First, genetic and epigenetic mutations in oncogenes (e.g., RAS and MYC) and tumor suppressor genes (e.g., TP53 and BRCA1) are shown in the initiation step. The second step shows the illustrated mechanisms like evasion of apoptosis, sustained angiogenesis, and immune evasion within the tumor microenvironment. Finally, visualize invasion and metastasis processes, with tumor cells spreading through the blood or lymphatic systems in the metastasis step. The diagnostic tools are highlight imaging techniques (e.g., CT, MRI, and PET) and molecular diagnostics (e.g., biomarkers and liquid biopsies) as they aid early detection and monitoring. Therapeutic interventions involve various therapeutic strategies. One is traditional therapies that include surgery, chemotherapy, and radiation therapy. Another is targeted therapies that include tyrosine kinase inhibitors and monoclonal antibodies. The other is immunotherapy that includes immune checkpoint inhibitors and CAR T-cell therapy.

The landscape of cancer diagnostics and therapy has undergone tremendous advancements in recent decades. From traditional surgery and chemotherapy to novel approaches, such as immunotherapy and gene editing, the fight against cancer is more hopeful than ever. However, challenges such as tumor heterogeneity, drug resistance, and accessibility to advanced treatments persist. The integration of personalized medicine combined with ongoing research in cancer biology and innovative technologies promises to transform cancer care and offer better outcomes for patients globally (Figure 2).

## Data Availability

Not applicable.
